# Bidirectional Retrieval of a Fractured Femoral Venous Sheath

**DOI:** 10.1016/j.jaccas.2026.108492

**Published:** 2026-05-22

**Authors:** Binbin Luo, Longfu Jiang, Hao Wu, Xianfeng Du

**Affiliations:** Department of Cardiology, Ningbo No. 2 Hospital, Wenzhou Medical University, Ningbo, Zhejiang, China

**Keywords:** atrial fibrillation, bidirectional traction, femoral venous sheath fracture, foreign body retrieval, snare

## Abstract

**Objective:**

To describe a step-by-step bailout technique for percutaneous retrieval of a fractured femoral venous sheath during redo atrial fibrillation ablation.

**Key Steps:**

1) Promptly recognize sheath hub-body separation and preserve the original J-shape guidewire within the retained sheath lumen; 2) confirm fragment location in the femoral vein by contrast venography through a second ipsilateral 8-F sheath in the right anterior oblique 20° view; 3) advance an SeQure LT-SP snare through the second sheath to capture and externalize the distal guidewire; 4) apply gentle simultaneous traction from both externalized wire ends to align the fragment with the original venous tract and remove it safely.

**Potential Pitfalls:**

Blind pulling before confirming fragment position may increase the risk of central embolization. This technique may be unsuitable if no guidewire remains within the retained fragment lumen, if the fragment embolizes centrally, or if major venous injury is suspected.

**Take-Home Messages:**

Preservation of the original intraluminal guidewire is the key prerequisite for this bailout strategy. Snare-assisted guidewire externalization with controlled bidirectional traction may allow safe percutaneous retrieval and avoid surgical cutdown.

## Visual Summary

**Figure:**
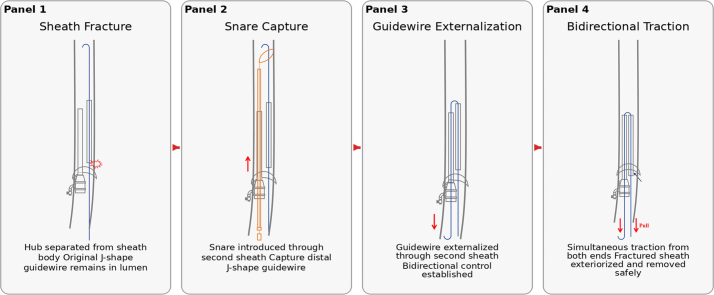

Catheter ablation is a guideline-supported therapy for symptomatic atrial fibrillation (AF).^1^ Standard electrophysiology procedures routinely use femoral venous introducer sheaths, but device failure with hub-body separation is exceedingly rare. A retained intravascular sheath fragment can embolize to the right heart or pulmonary arteries, posing a serious risk. Prompt percutaneous retrieval of foreign bodies is therefore indicated.^2^^,^^3^ Snare-based techniques are commonly used for foreign-body retrieval, but may be limited when fragments have no free end for grasping.^2^, ^3^, ^4^ In the present case, the novelty did not lie in a single isolated maneuver, but rather in the combination of several bailout steps used in sequence: preservation of the original guidewire within the retained sheath lumen, maintenance of ipsilateral secondary venous access, snare-assisted wire externalization, and controlled bidirectional traction retrieval. We describe a step-by-step bidirectional guidewire-assisted technique for controlled retrieval of a fractured femoral venous sheath fragment during redo AF ablation.

**Take-Home Messages**

• Sheath fracture during electrophysiology procedures should be recognized immediately, and the original intraluminal guidewire should be preserved whenever possible.
• A stepwise bailout strategy consisting of fluoroscopic confirmation, ipsilateral secondary venous access, snare-assisted wire externalization, and controlled bidirectional traction may allow safe percutaneous retrieval while avoiding surgical cutdown.

## Case Summary

A 53-year-old man was admitted with rapid, persistent AF and uncontrolled ventricular rate associated with recurrent chest tightness and exertional dyspnea. His medical history was notable for rheumatic heart disease and prior left atrial appendage occlusion plus AF ablation 5 years earlier. Because of recurrent symptomatic AF with poor ventricular rate control, redo catheter ablation was planned. Preprocedural anticoagulation consisted of low-molecular-weight heparin 4,250 international units (IU) once daily by subcutaneous injection. The patient was obese and had a history of right lower-extremity venous puncture 1 year earlier. These factors may have contributed to a deeper puncture tract, a steeper puncture angle, local fascial resistance, and greater difficulty in sheath withdrawal. Under local anesthesia, two 8-F sheaths were placed in the right femoral vein. During sheath exchange, resistance was encountered while withdrawing one 8-F introducer sheath. The sheath suddenly failed with hub-body separation, leaving the sheath body retained within the femoral venous tract. Initial fluoroscopy suggested retention of the fragment, and contrast venography through the second standard 8-F sheath in the right anterior oblique 20° view confirmed that the fractured sheath body remained in the femoral vein. Importantly, the original J-shape guidewire remained within the lumen of the retained sheath body, providing an immediate intraluminal rail for controlled retrieval. Because of concern for thrombosis around the retained foreign body, 3,000 IU heparin was administered through the second sheath before the retrieval maneuver. Surgical backup was available on standby throughout the procedure.

**Figure 1:**
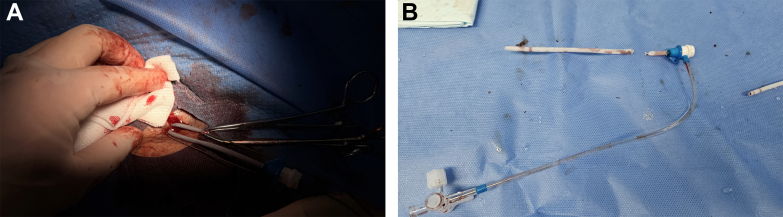
Fractured Femoral Venous Sheath With Hub-Body Separation (A) The retained sheath fragment is exteriorized and retrieved at the femoral venous access site using forceps after controlled traction and exposure. (B) Ex vivo view of the fractured 8-F introducer sheath demonstrating separation of the proximal hub from the sheath body, consistent with hub-body failure during withdrawal.

**Figure 2:**
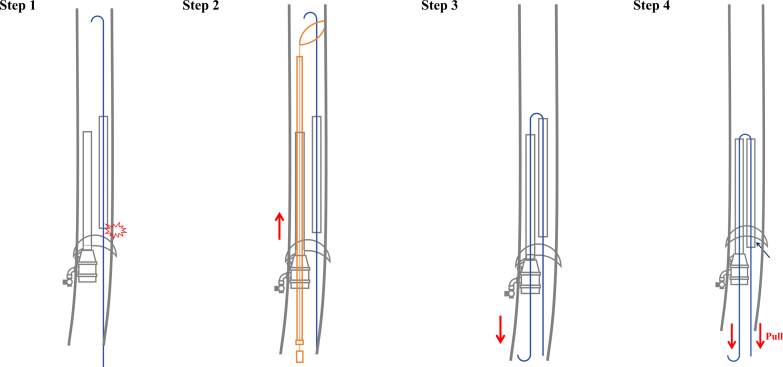
Stepwise Bidirectional Guidewire-Assisted Retrieval Technique Step 1: the retained sheath body remains within the femoral venous tract while the original J-shape guidewire is preserved within the sheath lumen. Step 2: a snare is advanced through a second ipsilateral femoral venous sheath to capture the distal guidewire segment. Step 3: the guidewire is externalized through the second sheath to establish bidirectional control. Step 4: simultaneous traction from both ends exteriorizes the sheath fragment for removal.

The retrieved fragment and separated device components are shown in Figures 1A and 1B. The technique is summarized in the Central Illustration, and the stepwise schematic is shown in Figure 2.

## How We Did It (Step-by-Step Technique)

### Step 1: preserve the original guidewire

The original J-shape guidewire was intentionally preserved within the lumen of the retained sheath fragment. This was recognized as the key prerequisite for the bailout strategy because it stabilized the fragment, reduced the risk of distal embolization, and provided a rail for subsequent retrieval.

### Step 2: confirm the retained fragment position

Contrast venography through the second ipsilateral 8-F femoral venous sheath in the right anterior oblique 20° projection confirmed that the fractured sheath body remained in the femoral vein and had not embolized centrally. We intentionally avoided blind pulling or aggressive manipulation before confirming fragment position in order to reduce the risk of central migration. The J-shaped segment or a segment that can be looped securely was targeted (Figure 2, step 2).

### Step 3: capture and externalize the guidewire

An LT-SP snare (25 mm loop diameter, 65 cm length) was advanced through the second ipsilateral femoral venous sheath under fluoroscopic guidance to capture the distal portion of the retained J-shape guidewire. The J-shaped segment, or any segment that could be securely looped, was targeted. Once snared, the guidewire was withdrawn through the second sheath until its distal end was externalized, thereby establishing true bidirectional control of the wire-fragment system (Figure 2, step 3; Central Illustration).

### Step 4: apply simultaneous bidirectional traction

Gentle, simultaneous traction was applied from both externalized guidewire ends. This aligned traction vectors along the original venous access tract, enabling controlled proximal movement of the retained sheath body (Figure 2, step 4; Central Illustration).

### Step 5: exteriorize the sheath fragment

Coordinated traction was continued until the proximal end of the sheath body emerged at the venous entry site. Abrupt pulling was avoided to prevent vascular injury.

### Step 6: grasp and remove the fragment

Once visible externally, the sheath fragment was grasped with forceps and removed completely through the original access site (Figure 1A). The ablation procedure was then continued as clinically indicated.

## Case Outcome and Follow-Up

The retained sheath fragment was successfully retrieved percutaneously without surgical cutdown. The original access site remained usable, and the planned AF ablation was completed successfully after re-establishing sheath access over the guidewire. The patient remained hemodynamically stable throughout the remainder of the procedure. Postprocedural follow-up included venous ultrasound at 24 hours, which showed no hematoma and no venous thrombosis. No delayed access-site discomfort or other vascular complications were observed during the early recovery period.

## Discussion

This case highlights a rare but potentially serious vascular access complication during AF ablation. Acute complications of AF ablation have been systematically reported, emphasizing the importance of recognizing and managing procedure-related adverse events.^5^ In this patient, several anatomical and procedural factors may have contributed to hub-body separation of the sheath. These include obesity with a deeper puncture tract and steeper puncture angle, previous ipsilateral puncture history, and possible local tissue-related resistance during sheath withdrawal.

Mechanistically, several explanations may account for the resistance that preceded sheath fracture. First, tissue entrapment around the sheath tip or shaft may have created localized fixation. Second, fascial resistance within the puncture tract may have increased withdrawal force, especially in the setting of obesity and a deeper access tract. Third, sheath kinking may have developed during manipulation or withdrawal, particularly if the puncture angle was steep or the tract was tortuous. The interaction of these factors may have increased stress at the junction between the sheath hub and body, eventually leading to separation.

Conventional retrieval options include surgical cutdown or endovascular snaring of the fragment.^2^, ^3^, ^4^ Surgical access is definitive but more invasive. Direct snaring of the fragment may fail if no portion is free to grasp and may inadvertently advance the fragment.

The novelty of the present technique lies in the combination of 4 bailout elements: preservation of the original intraluminal guidewire, maintenance of ipsilateral secondary venous access, snare-assisted distal wire externalization, and controlled bidirectional traction retrieval. Although each component is conceptually familiar, the stepwise integration of these measures created a practical rescue strategy for this specific scenario.

## Advantages of the Present Approach

First, preserving the guidewire within the sheath lumen provides intrinsic stabilization and a controlled “rail,” minimizing uncontrolled fragment movement. Second, snare-assisted guidewire externalization creates true bidirectional control. Third, simultaneous traction aligns forces along the original access tract, facilitating controlled exteriorization without additional vascular exposure. This strategy leverages readily available electrophysiology laboratory tools and is reproducible for operators without specialized retrieval devices beyond a standard snare.

## Limitations of the Technique

This approach may not be applicable if no guidewire remains within the retained fragment lumen, if the fragment embolizes centrally, if severe kinking prevents coaxial alignment with traction, or if venous perforation or major vessel injury is suspected. In such situations, alternative endovascular retrieval strategies or surgical intervention may be more appropriate.

## Conclusions

Femoral venous sheath hub-body separation is rare but potentially serious. When the original guidewire remains preserved within the retained sheath lumen, snare-assisted bidirectional retrieval provides a simple, controlled, and reproducible percutaneous bailout strategy that may avoid surgical cutdown.

**Visual Summary:**
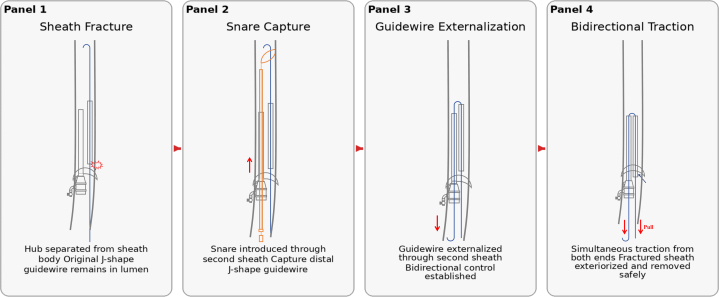
Bidirectional Guidewire-Assisted Retrieval Technique After hub-body separation of a femoral venous sheath during atrial fibrillation ablation, the sheath body remained retained in the femoral vein while the original J-shape guidewire was preserved within the sheath lumen. A snare introduced through a second ipsilateral femoral venous sheath captured the distal guidewire and externalized it, establishing bidirectional wire control. Simultaneous traction applied from both ends enabled controlled exteriorization and safe removal of the fractured sheath fragment through the original access site.

## Funding Support and Author Disclosures

The original study was supported by the Project of Ningbo Leading Medical & Health Discipline, China (2023Z191), and Ningbo Major Research and Development Plan Project (2024Z235). The funders had no role in study design, data collection and analysis, decision to publish, or preparation of the manuscript. The authors have reported that they have no relationships relevant to the contents of this paper to disclose.
